# Potential virulence of *Klebsiella* sp. isolates from enteral diets

**DOI:** 10.1590/1414-431X20154316

**Published:** 2015-07-10

**Authors:** S.C.L. Pereira, M.C.D. Vanetti

**Affiliations:** 1 Departamento de Nutrição, Escola de Enfermagem, Universidade Federal de Minas Gerais, Belo Horizonte, MG, Brasil; 2 Departamento de Microbiologia, Universidade Federal de Viçosa, Viçosa, MG, Brasil

**Keywords:** Klebsiella, Enteral diets, Pathogenicity

## Abstract

We aimed to evaluate the potential virulence of *Klebsiella* isolates from enteral diets in hospitals, to support nosocomial infection control measures, especially among critical-care patients. Phenotypic determination of virulence factors, such as capsular expression on the external membrane, production of aerobactin siderophore, synthesis of capsular polysaccharide, hemolytic and phospholipase activity, and resistance to antibiotics, which are used therapeutically, were investigated in strains of *Klebsiella pneumoniae* and *K. oxytoca*. Modular industrialized enteral diets (30 samples) as used in two public hospitals were analyzed, and *Klebsiella* isolates were obtained from six (20%) of them. The hypermucoviscous phenotype was observed in one of the *K. pneumoniae* isolates (6.7%). Capsular serotypes K1 to K6 were present, namely K5 and K4. Under the conditions of this study, no aerobactin production, hemolytic activity or lecithinase activity was observed in the isolates. All isolates were resistant to amoxicillin and ampicillin and sensitive to cefetamet, imipenem, chloramphenicol, gentamicin and sulfamethoxazole-trimethoprim. Most *K. pneumoniae* isolates (6/7, 85.7%) from hospital B presented with a higher frequency of resistance to the antibiotics tested in this study, and multiple resistance to at least four antibiotics (3/8; 37.5%) compared with isolates from Hospital A. The variations observed in the antibiotic resistance profiles allowed us to classify the *Klebsiella* isolates as eight antibiotypes. No production of broad-spectrum β-lactamases was observed among the isolates. Our data favor the hypothesis that *Klebsiella* isolates from enteral diets are potential pathogens for nosocomial infections.

## Introduction

Bacteria of the genus *Klebsiella* frequently cause nosocomial infections and are associated with high morbidity and mortality in people (1). *K. pneumoniae*, in particular, is the most clinically important *Klebsiella* species and occurs in nosocomial infections, such as those of the urinary tract, and in cases of pneumonia and septicemia (1).

Given the severity of the worldwide situation regarding nosocomial infections, control efforts should be directed at identifying the sources of contamination and means of transmission to implement preventive measures (2). The gastrointestinal tract and the hands of hospital medical staff are recognized as sources of contamination by *Klebsiella* (3,4). These bacteria are also found frequently in enteral diets. However, the involvement of such foods as sources of this opportunistic pathogen has received little attention in the scientific and medical literature (5,6).

Recently, a study identified *K. pneumoniae* as the nosocomial pathogen responsible for a large-scale outbreak, which occurred via the hospital’s own food supply chain (7). This was the first study to address the transmission of multidrug-resistant *Klebsiella* through food in the hospital environment. Furthermore, according to Podschun et al. (8) *K. pneumoniae* isolates from nonclinical sources are, in common with clinical isolates, able to express virulence factors. Among the factors that contribute to the pathogenicity of *Klebsiella*, the nature of the polysaccharide capsule of the external membrane, production of siderophores and fimbrial adhesins, and synthesis of capsular polysaccharide (as characterized by the mucoid appearance of the colonies) are considered to underlie this virulence process (8-11).

Other factors, such as hemolysin and phospholipase production, as well as multidrug resistance against the antibiotics used for therapy, are considered to be intensifiers of the virulence potential of *Klebsiella* (9). Moreover, the number of outbreaks of nosocomial infections caused by multidrug-resistant *Klebsiella* isolates, especially extended-spectrum beta-lactamase (ESBL) producers, have increased over the last few years (12,13).

Despite this, no studies on enteral diets similar to the study described herein were found in the scientific literature we consulted. Therefore, the objective of the present study was to investigate the pathogenicity of the *Klebsiella* strains isolated from modular enteral diets, by analyzing their phenotypic expression of virulence factors and their antibiotic resistance profiles.

## Material and Methods

### Origin of the isolates

Modular industrialized (milk-based and food supplement) enteral diets (30 samples) as used in two public hospitals in the State of Minas Gerais (Brazil) were analyzed, and *Klebsiella* sp. isolates were obtained from six (20%) of them. The isolates were analyzed for their potential virulence and the results are reported in Table 1 **.** We emphasize that the samples were collected before administration to the patients.

**Table:**
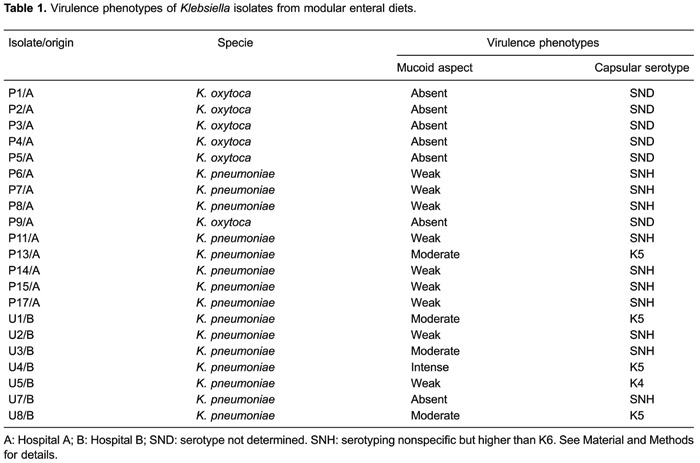


### Phenotypic determination of virulence factors

*Mucoid appearance, presence of capsules, and serotyping*. The mucoid appearance of the colonies was observed after incubation (37°C, 48 h) of the *Klebsiella* isolates in brain heart infusion (BHI) agar (Difco Laboratories, USA). Individual colonies were tested for their capacity to form viscous chains. Chain formation (length &5 mm) was an indicator of a positive phenotype for hypermucoviscosity (14).

The presence of capsules in the *Klebsiella* isolates was observed on slides treated with alcoholic Congo red (0.2% solution; SRL Chemicals, India) using an optical microscope (oil immersion lens; Leica DMLS, Leica Microsystems, Germany). The unstained region seen on a blue background around the red central nucleus of the bacterial cells indicated the presence of a capsule (10). Additionally, the *K. pneumoniae* isolates were sent to the Laboratório de Enterobactérias, Departamento de Bacteriologia, Fundação Oswaldo Cruz, Rio de Janeiro, RJ, Brazil for immunological analysis of the serotypes with regard to the K1 to K6 capsular antigens of the external membrane, as determined by antigen-antibody reaction analysis.

*Analysis of aerobactin production*. Aerobactin production was demonstrated by using the LG 1522 strain of *Escherichia coli* as an indicator. This mutant is deficient in aerobactin synthesis, but has the aerobactin receptor (11). This bacterium was cultivated in Petri dishes containing M9 agar, with addition of 200 µM 2,2′-dipyridine (Sigma Chemical Co., USA). One colony of each *Klebsiella* isolate to be tested was inoculated into the agar medium containing the indicator strain. Aerobactin-producing strains were viewed as a halo of LG 1522 *E. coli* cell growth around the inoculated isolates. The *E. coli* strain LG 1315, which is genotypically (*ara entA lac leu mtL proC rpsL supE thi tonA trpE xyl*) and phenotypically certified as an aerobactin producer, was the positive control. The *E. coli* strain HB101, which is certified as an aerobactin non-producer strain, was the negative control (15).

*Phosphatidylcholine-specific phospholipase C (PC-PLC)*. PC-PLC activity was determined using the method described by Coffey et al. (16). *Klebsiella* cultures were grown in BHI broth (4 mL; Difco Laboratories) and incubated at 37°C for 18-24 h. Aliquots (3 µL) from each culture were inoculated onto the surface of BHI agar containing 2% NaCl and 5% egg yolk emulsion, previously diluted in 0.85% NaCl (1:1, v/v). The plates, incubated (37°C, 96 h) in a Gas-Pak jar with an anaerobiosis generator (AnaeroGen™, Oxoid, UK), were observed to determine whether a lecithin hydrolysis halo around the colonies was present.

*Hemolysin activity*. The hemolytic activity of the *Klebsiella* cultures was investigated using blood agar, as described by Kokosharov and Phetisova (17). BHI plates (Oxoid) containing 5% defibrinated rabbit blood were prepared and small wells (3 mm) were produced. The wells were used to inoculate 5 µL bacterial suspensions, after which the plates were incubated at 37°C. The plates were observed after anaerobic incubation at 37°C for 24 h and the presence of a partial halo or total hemolysis was considered a hemolysin-positive result.

### Antibiotic susceptibility

The susceptibility of each *Klebsiella* isolate to antimicrobial agents was determined through the disk diffusion method in Mueller-Hinton agar (Difco Laboratories), according to the guidelines of the Clinical and Laboratory Standards Institute (CLSI) (18). The antibiotics (Sensibiodisc-CECON, Brazil) used in this study were as follows: nalidixic acid (NAL, 30 µg), amikacin (AMI, 30 µg), amoxicillin (AMO, 10 µg), amoxicillin-clavulanic acid (AMC, 30 µg/10 µg), ampicillin (AMP, 10 µg), chloramphenicol (CLO, 30 µg), gentamicin (GEN, 10 µg), imipenem (IMP, 30 µg), kanamycin (KN, 30 µg), neomycin (NO, 30 µg), tetracycline (TET, 30 µg), ticarcillin-clavulanic acid (TIC, 75 µg/10 µg), and trimethoprim-sulfamethoxazole (SUT, 1.25/23.75 µg). To identify ESBL-producing strains, the cultures were screened with the following antibiotics: cefotaxime (CTX, 30 µg), ceftazidime (CAZ, 30 µg), cefetamet (CEF, 10 µg), cefalotin, (CFL; 30 µg), and aztreonam (ATM, 30 µg), according to the CLSI guidelines (18). After incubation (35°C, 16-18 h) with cefetamet, ceftazidime, aztreonam, cefotaxime, and cefalotin, the isolates with growth-inhibition halos were suspected to be ESBL-producing strains. The E test (AB BIODISK, Sweden) was used as a confirmatory test, and ceftazidime and cefotaxime antibiotics were used as substrates. Reduction in the minimum inhibitory concentration (MIC) of the antimicrobial agent in association with the beta-lactamase inhibitor was compared with the MIC of the antimicrobial agent beta-lactamase alone (ceftazidime or cefotaxime) and was indicative of ESBL-producing strains.

## Results

We analyzed 30 samples from the diets used in public hospitals and identified 21 *Klebsiella* isolates. Most (n=15; 71.4%) of them were of the *K. pneumonia* species. The *K. oxytoca* species was identified in one of the hospitals (Table 1).

Most (42.9%) of the *Klebsiella* isolates obtained from the modular enteral diets formed colonies with a low mucoid appearance in BHI agar. Four isolates (26.7%) of *K. pneumoniae* had a moderate mucoid appearance and only the isolate U4 (6.7%) presented a hypermucoviscosity phenotype. The presence of a capsule in the isolate U4 and isolates with colonies of moderate mucoid appearance were confirmed by observing the preparations under a microscope. In isolates whose colonies presented a low mucoid appearance, no capsule could be observed through the staining used in this study. Additionally, five isolates with the presence of capsule antigens between K1 and K6 were identified (Table 1).

**Figure 1:**
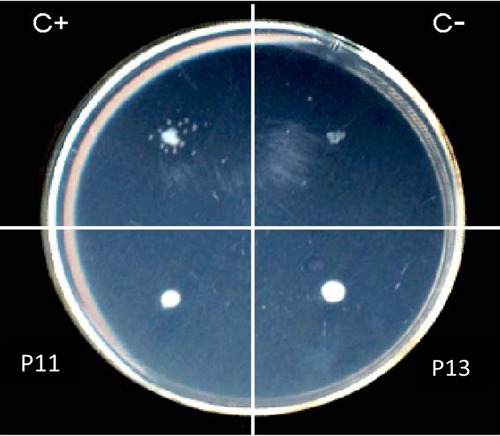
Aerobactin production on M9 agar with 200 µg 2,2'-bipyridine. A positive test is indicated by the presence of satellite colonies around the isolate, as seen in the positive control (C+). C-: negative control. P11 and P13 isolates are described in Table 1.

Under our experimental conditions, the *Klebsiella* isolates from the enteral diets we tested did not exhibit aerobactin production (Figure 1), hemolytic activity, or lecithinase activity.

All the *Klebsiella* isolates (21) from the enteral diets were resistant to amoxicillin and ampicillin (Table 2). Six isolates (28.6%) were shown to be resistant when amoxicillin was used in association with the beta-lactamase inhibitor, clavulanic acid (Table 2). This behavior was also observed in relation to the association between ticarcillin and clavulanic acid, albeit less frequently (Table 2).

**Table:**
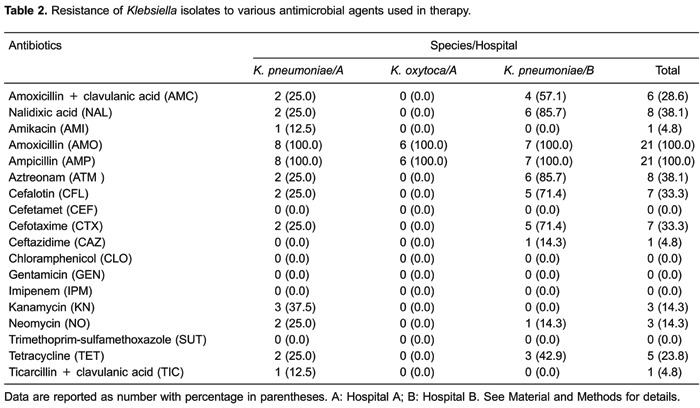


The *Klebsiella* isolates were sensitive to beta-lactamic group antibiotics, namely cefetamet, which is a third-generation cephalosporin, and imipenem, which is a carbapenem antibiotic (Table 2). These isolates were also inhibited by different groups of antibiotics, such as chloramphenicol (a quinolone), gentamicin (an aminoglycoside), and sulfatrim (a mixture of trimethoprim and sulfamethoxazole).

The *K. pneumoniae* isolates found in the enteral diets from hospital B showed a high frequency of resistance to the antibiotics tested and multiple resistance to at least four antibiotics compared with the isolates from hospital A (Table 2).

The differences observed in the antibiotic resistance profiles among the *Klebsiella* isolates allowed us to classify them into the antibiotypes described in Table 3.

**Table:**
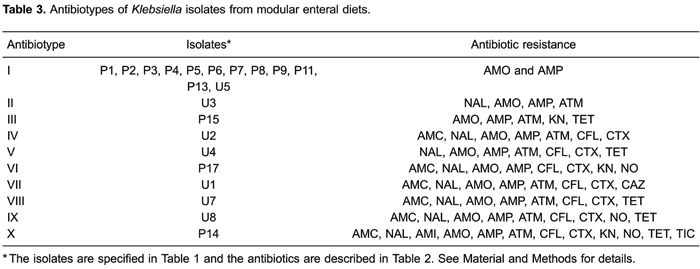


ESBL production by the *K. pneumoniae* and *K. oxytoca* isolates was not observed. This observation could be made because no reduction was seen in the MIC of the antimicrobial agent in association with the beta-lactamase inhibitor in comparison with that of the beta-lactamic antimicrobial agent alone (ceftazidime or cefotaxime). Resistance to these antibiotics, which were used to detect ESBL-producing strains, was in the range 0-38.1% (Table 2). The highest frequencies of resistance were observed for the beta-lactamic agents, aztreonam (38.1%) and cefotaxime (33.3%), whereas the lowest frequencies of resistance were recorded for ceftazidime (4.8%) and cefetamet (0%) (Table 2).

## Discussion

In this study, we found the frequency of the hypermucoviscous phenotype among the colonies formed by *K. pneumonia* isolates to be low (6.7%). However, this finding deserves further scrutiny. This is because some studies conducted on clinical samples have reported a low frequency for this phenotype. For example, Vernet et al. (19) observed that only 7.0% of 241 *K. pneumoniae* clinical isolates had this phenotype. In a study with greater sample coverage (20), 455 *K. pneumoniae* clinical isolates were found to be involved in consecutive cases of bacteremia in seven countries. In two of these countries, a very high frequency of the hypermucoviscous phenotype was found in isolates that caused pneumonia (94%) and invasive syndrome (100%). However, in other countries the mean frequency was 2% (i.e., a lower frequency than that found in the present study).

The mucoid appearance of *Klebsiella* colonies is related to the production of a capsule, a virulence factor essential for *Klebsiella* to overcome a host’s immune system (21). Furthermore, a recent study attributed the role of the *magA* and *rmpA* genes as determinants of the hypermucoviscous phenotype in the pathogenesis of the K1 capsular serotype of *K. pneumoniae,* which is involved in bacteremia (22). The *rmpA* gene, which codes for a positive regulator of extracapsular mucopolysaccharide expression (20), was also identified in two (2/54) clinical isolates out of a total of five isolates with the hypermucoviscous phenotype. However, the isolates were not serotyped for K1-K6 positivity (9). The hypermucoviscous phenotype is seen predominantly in strains with the K1 and K2 serotypes; these are the clinical isolates causing invasive diseases. This would explain the low hypermucoviscosity rates found among the isolates in the present study (9).

The presence of a capsule is an important virulence factor relating to the severity of an infection. A capsule is generally present in clinical strains, and 78 capsule types were identified in *K. pneumoniae*. However, because of the limitations of traditional serotyping methods, capsule type determination is often difficult (23). In the present study, isolates were found to have the K5 (4) and K4 (1) serotypes out of a possible range of six main serotypes (K1-K6) for this microbial species. Among the 78 distinct capsule antigens of *K. pneumoniae*, only the K1, K2, and K5 antigens are considered highly virulent, being frequently associated with severe infections in humans and animals. One of these types of antigen was found in four of the isolates in this study.

None of the isolates we tested exhibited aerobactin production. According to some authors (8,9,19), *Klebsiella* isolates with aerobactin-positive phenotypes are rare, irrespective of the species or isolation source.

Aerobactin is one of the siderophores (extracellular ferric chelating agents) secreted by bacterial cells; hence, it can eliminate free Fe^3+^ ions from iron-binding proteins in the host (9). These siderophores play a critical role in bacterial virulence. Based on our results and the information found in the scientific literature (19), *Klebsiella* should be included in the group of enterobacteria that present low aerobactin production rates (<20%). In this regard, and given that iron bioavailability in the host is extremely low (10^-18^ M), iron is essential for microbial growth, and the incidence of the aerobactin phenotype among *Klebsiella* isolates is low, we raise the question of which mechanisms in the host would ensure iron supply to these bacteria (8,9,19).

It has been shown that enterobactin, another ferric chelating agent, is present in most clinical isolates of *K. pneumoniae* (9,15). The role of enterobactin in *Klebsiella* virulence is still uncertain, but expression of the enterobactin gene seems to be activated during infection. A recent study found the presence of genes that code for synthesis of enterobactin in clinical isolates of noninvasive infections (bacteremia) (9).

The absence of hemolytic activity found under the conditions of this study, which are often not reproducible under *in vitro* conditions, may be related to a lack of activators for expression of genes that code for this enzyme (24). The hemolysin virulence factor was first detected by Albesa (1989) in a test in which blood agar containing rabbit erythrocytes was used (24). Since then, this investigator and her group have been studying this cytolysin, and its specificity for rabbit erythrocytes has been confirmed. Hemolysin is a pore-forming toxin that makes some nutrients, such as the iron ion in hemoglobin, available. In a recent study, none of the 54 clinical isolates presented with hemolysis or the presence of the *hlyA* and *cnf-1* genes encoding this activity. This is consistent with the lack of studies reporting the genotypic and phenotypic presence of hemolysis in *Klebsiella*, although hemolytic activity has been described (24,25). Lecithinase or PC-PLC activity was absent in all isolates under the conditions in which this was determined. This result is in agreement with that of Singh et al. (26) who reported that lecithinase activity in *Klebsiella* isolates was rare (only 2.4% of their 168 clinical isolates of *K. pneumoniae* presented such activity). Also, they found that lecithinase activity was absent among the 11 isolates of *K. oxytoca*. Their study was the first report on lecithinase activity in *Klebsiella* isolates, and the role of this virulence factor in the pathogenicity of this genus of bacteria has not yet been fully clarified.

Regarding the antibiotics used in the present study, a high prevalence of resistance to ampicillin and amoxicillin was observed. Resistance to ampicillin was also observed in a study on clinical isolates of *K. pneumonia* (27) and in a study on isolates of this enterobacterium in expressed human breast milk (28).

Additionally, the prevalence of resistance to multiple antibiotics among isolates from hospital B (85.7%) was higher than among isolates from hospital A (37.5%). According to Davies (27), the prevalence of antibiotic resistance between different hospitals may vary because of local epidemiological factors such as control of the use of antibiotics and the preventive measures applied by hospital infection committees in relation to multidrug resistant strains. Therefore, strategies to prevent nosocomial emergence and spread of antibiotic-resistant organisms are essential. In this sense, improvement in hospital infection control practices outlined for prevention and transmission of multidrug resistant microorganisms is necessary, especially in hospital B. A combination of epidemiological surveillance, molecular genotyping, observational studies on compliance, and mathematical modeling will improve our ability to determine the quantitative effects of individual infection control measures. This may help us to design more effective infection control programs (29-32).

Here, no correlation between the high incidence of resistance to cefotaxime and ESBL production was observed. This is an important clinical result, although the isolates were multiply resistant to up to 12 antibiotics.

The condition of multidrug resistance observed in the present study justifies the assertion that bacterial resistance to antimicrobial agents is a serious public health problem. This is the real situation within which control initiatives have been proposed. In Brazil, legally established control over medications based on antimicrobial agents for use under medical prescription, either singly or in combination, was initiated in 2010. Thus, these substances can now be dispensed only through a specially controlled prescription (33).

Moreover, the observation that *Klebsiella* isolates are resistant to multiple antibiotics has significant clinical importance. This is because enteral diets are prescribed for patients who are highly susceptible to nosocomial infections and are concentrated in intensive care units. Such patients are subject to the intrinsic risks related to complex underlying diseases, nutritional vulnerability, extreme ages (premature infants and elderly people), and the effects of drug treatments, in addition to extended-spectrum antimicrobial agents. Furthermore, given that hospitals have complex organizational structures, patients are subject to extrinsic risks such as prolonged stays in hospital environments and routine invasive procedures, the quality of which is not guaranteed for all hospital services (12). In this context, the risk to health becomes more severe because this environment is a potential source of genes for resistance to pathogenic microorganisms, especially those that cause highly toxic infections. Additionally, the intestinal tract is an appropriate niche for transference of genes related to resistance (34).

Some authors have suggested that transfer of plasmids that carry more than one antimicrobial resistance gene facilitates horizontal gene transmission of different resistance mechanisms to other bacteria, thus further worsening the existing problem of multiple resistance to antibiotics in hospital environments (27,35). Such transfers are probably more effective when hosts are simultaneously subjected to selective pressure from an antimicrobial substance to which the microorganisms involved are resistant. From this perspective, hospitals are environments where drug-resistant microorganisms and others that determine such resistance are more likely to be present. Thus, in such environments, horizontal gene transfer has a greater chance of occurring and the consequences may be more severe in terms of therapeutic failure (36,37).

The occurrence of chromosomal mutations alone does not explain the rapid acquisition of genetic resistance to antibiotics in bacteria. It is established that plasmids are particularly important in the evolution of antibiotic-resistant bacteria. It has also been suggested that transfer of plasmids carrying resistance genes facilitates horizontal gene transmission to other bacteria (23,27). Food seems to be an effective source for human acquisition of resistant bacteria and genes for resistance to drugs, but the extent and real consequences of this type of exposure have not yet been fully investigated (38).

The data obtained in this study favor the hypothesis that *Klebsiella* isolates from enteral diets are potential pathogens for nosocomial infections, especially in critical-care patients. This is because capsular serotypes, which are only identified in severe cases of nosocomial infection, were observed among the isolates in our study. They include the K5 and K4 serotypes, which are among the serotypes of clinical importance (K1 to K6). Additionally, the presence of the hypermucoviscous phenotype was observed, thus increasing the probability of an invasion process occurring in cases of isolates with one of these important capsular serotypes. This profile of virulence, which may provide a partial explanation for infection by *Klebsiella*, is a risk for microbiological security. This situation occurs during propagation of infection in intensive care units, where the intrinsic and extrinsic risks favor nosocomial infections. The synergy that may exist between *Klebsiella* virulence factors should be highlighted, especially when it occurs in combination with resistance to the multiple types of antibiotic used for therapeutic use, as has also been shown in the present study. Further studies are necessary to assess the pathogenicity of *Klebsiella* isolates under *in vivo* conditions.

The present results also showed that control measures against nosocomial infections are important for those handling enteral diets. Regarding individual and collective practices, participation of all sectors of the hospital in the control of this situation is necessary. We emphasize that rigorous and systematic monitoring of the risks related to the origin of the food has to be applied to the hospital environment. Good food-handling practices are necessary to control the dissemination of *Klebsiella* and other bacteria in the hospital environment.

In this regard, our study adds new knowledge that supports preventative strategies against hospital acquired infections caused by *Klebsiella* and emphasizes the importance of measures linking clinical outcomes with the enteral nutrition sector where relevant to do so. Our findings are in agreement with our aim of strengthening the interfaces between infection control and preventive actions and also between these controls and the promotion of quality within all healthcare services. The present study also endorses the need to direct healthcare funding policies towards implementation of risk control and prevention measures in strategic sectors of the healthcare services. Reflection on all the possible strategies that might contribute towards changing the current panorama of nosocomial infections, with investments in research and infection control updates, is necessary. We emphasize that data on nosocomial infections (as described in the present study and other recent studies) should be disseminated routinely to all healthcare professionals, and to hospital administration staff.

## References

[1] Lin YT, Wang FD, Chan YJ, Fu YC, Fung CP (2014). Clinical and microbiological characteristics of
tigecycline non-susceptible *Klebsiella pneumoniae* bacteremia in
Taiwan. BMC Infect Dis.

[2] WHO. World Health Organization (2011). Core components for infection prevention and control programs.
Assessment tools for IPC programmes. Prevention of hospital-acquired
infections..

[3] Ekrami AR, Kayedani A, Jahangir M, Kalantar E, Jalali M (2011). Isolation of common aerobic bacterial pathogens from the
environment of seven hospitals, Ahvaz, Iran. Jundishapur J Microbiol.

[4] Westerbeek EA, van den Berg A, Lafeber HN, Fetter WP, van Elburg RM (2011). The effect of enteral supplementation of a prebiotic
mixture of non-human milk galacto-, fructo- and acidic oligosaccharides on
intestinal permeability in preterm infants. Br J Nutr.

[5] Hurrell E, Kucerova E, Loughlin M, Caubilla-Barron J, Hilton A, Armstrong R (2009). Neonatal enteral feeding tubes as loci for colonisation
by members of the Enterobacteriaceae. BMC Infect Dis.

[6] Kanevsky-Mullarky I, Nedrow AJ, Garst S, Wark W, Dickenson M, Petersson-Wolfe CS (2014). Short communication: comparison of virulence factors in
*Klebsiella pneumoniae* strains associated with multiple or
single cases of mastitis. J Dairy Sci.

[7] Calbo E, Freixas N, Xercavins M, Riera M, Nicolas C, Monistrol O (2011). Foodborne nosocomial outbreak of SHV1 and
CTX-M-15-producing *Klebsiella pneumoniae*: epidemiology and
control. Clin Infect Dis.

[8] Podschun R, Pietsch S, Holler C, Ullmann U (2001). Incidence of *Klebsiella* species in
surface waters and their expression of virulence factors. Appl Environ Microbiol.

[9] El Fertas-Aissani R, Messai Y, Alouache S, Bakour R (2013). Virulence profiles and antibiotic susceptibility
patterns of *Klebsiella pneumoniae* strains isolated from different
clinical specimens. Pathol Biol.

[10] Russo TA, Olson R, Macdonald U, Metzger D, Maltese LM, Drake EJ (2014). Aerobactin mediates virulence and accounts for increased
siderophore production under iron-limiting conditions by hypervirulent
(hypermucoviscous) *Klebsiella pneumoniae*. Infect Immun.

[11] Li W, Sun G, Yu Y, Li N, Chen M, Jin R (2014). Increasing occurrence of antimicrobial-resistant
hypervirulent (hypermucoviscous) *Klebsiella pneumoniae* isolates
in China. Clin Infect Dis.

[12] Viale P, Giannella M, Lewis R, Trecarichi EM, Petrosillo N, Tumbarello M (2013). Predictors of mortality in multidrug-resistant
*Klebsiella pneumoniae* bloodstream infections. Expert Rev Anti Infect Ther.

[13] Hawkey PM (2015). Multidrug-resistant Gram-negative bacteria: a product of
globalization. J Hospital Infec.

[14] Wiskur BJ, Hunt JJ, Callegan MC (2008). Hypermucoviscosity as a virulence factor in experimental
*Klebsiella pneumoniae* endophthalmitis. Invest Ophthalmol Vis Sci.

[15] Podschun R, Sievers D, Fischer A, Ullmann U (1993). Serotypes, hemagglutinins, siderophore synthesis, and
serum resistance of *Klebsiella* isolates causing human urinary
tract infections. J Infect Dis.

[16] Coffey A, Rombouts FM, Abee T (1996). Influence of environmental parameters on
phosphatidylcholine phospholipase C production in Listeria monocytogenes: a
convenient method to differentiate L. monocytogenes from other Listeria
species. Appl Environ Microbiol.

[17] Kokosharov T, Phetisova K (2002). Hemolysins and aerobactin in *Salmonella
callinarum* strains isolated from poultry. Rev Med Vet.

[18] CLSI - Clinical and Laboratory Standards Institute (2015). Performance standards for antimicrobial susceptibility testing.
Approved Standard M100-S25..

[19] Vernet V, Madoulet C, Chippaux C, Philippon A (1992). Incidence of two virulence factors (aerobactin and
mucoid phenotype) among 190 clinical isolates of *Klebsiella
pneumoniae* producing extended-spectrum beta-lactamase. FEMS Microbiol Lett.

[20] Yu VL, Hansen DS, Ko WC, Sagnimeni A, Klugman KP, von Gottberg A (2007). Virulence characteristics of Klebsiella and clinical
manifestations of *K. pneumoniae* bloodstream
infections. Emerg Infect Dis.

[21] Evrard B, Balestrino D, Dosgilbert A, Bouya-Gachancard JL, Charbonnel N, Forestier C (2010). Roles of capsule and lipopolysaccharide O antigen in
interactions of human monocyte-derived dendritic cells and *Klebsiella
pneumoniae*. Infect Immun.

[22] Cheng KC, Lee MF, Chuang YC, Yu WL (2015). First description of lung abscess caused by ST23 clone
capsule genotype K1 *Klebsiella pneumoniae*. J Formos Med Assoc.

[23] Pan YJ, Lin TL, Chen YH, Hsu CR, Hsieh PF, Wu MC (2013). Capsular types of *Klebsiella pneumoniae*
revisited by wzc sequencing. PLoS One.

[24] Albesa I (1989). Klebsiella pneumoniae. Klebsiella pneumoniae haemolysin adsorption to red blood
cells..

[25] Gundogan N, Citak S, Yalcin E (2011). Virulence properties of extended spectrum
beta-lactamase-producing *Klebsiella* species in meat
samples. J Food Prot.

[26] Singh BR, Sharma VD, Chandra R (1999). Detection, prevalence, purification and characterization
of lecithinase of *Klebsiella pneumoniae*. Indian J Exp Biol.

[27] Davies J, Davies D (2010). Origins and evolution of antibiotic
resistance. Microbiol Mol Biol Rev.

[28] Novak FR, Almeida JA, Asensi MD, Moraes BA, dos Prazeres RD (2001). [Antimicrobial resistance of coliform isolates from
expressed human milk]. Cad Saúde Pública.

[29] Freyne B, Carr J, Osowicki J, Steer A, Curtis N, Bryant PA (2015). Hospital-wide rollout of antimicrobial stewardship: a
stepped-wedge randomized trial. Clin Infect Dis.

[30] Eisenberg JN, Goldstick J, Cevallos W, Trueba G, Levy K, Scott J (2012). In-roads to the spread of antibiotic resistance:
regional patterns of microbial transmission in northern coastal
Ecuador. J R Soc Interface.

[31] Salles MJC, Zurita J, Mejía C, Villegas V (2013). Resistant Gram-negative infections in the outpatient
setting in Latin America. Epidemiol Infect.

[32] Bonten MJ, Austin DJ, Lipsitch M (2001). Understanding the spread of antibiotic resistant
pathogens in hospitals: mathematical models as tools for control. Clin Infect Dis.

[33] Brasil. Ministério da Saúde (2011). Resolução de Diretoria Colegiada (RDC) No. 20, de 5 de maio de
2011..

[34] Andremont A (2003). Commensal flora may play key role in spreading
antibiotic resistance. ASM News.

[35] Herrera-Leon S, Gonzalez-Sanz R, Herrera-Leon L, Echeita MA (2011). Characterization of multidrug-resistant
*Enterobacteriaceae* carrying plasmid-mediated quinolone
resistance mechanisms in Spain. J Antimicrob Chemother.

[36] Alekshun MN, Levy SB (2007). Molecular mechanisms of antibacterial multidrug
resistance. Cell.

[37] Evans HL, Lefrak SN, Lyman J, Smith RL, Chong TW, McElearney ST (2007). Cost of Gram-negative resistance. Crit Care Med.

[38] Machado E, Coque TM, Canton R, Sousa JC, Peixe L (2008). Antibiotic resistance integrons and extended-spectrum
{beta}-lactamases among Enterobacteriaceae isolates recovered from chickens and
swine in Portugal. J Antimicrob Chemother.

